# Antibiotic Resistance: Moving From Individual Health Norms to Social Norms in One Health and Global Health

**DOI:** 10.3389/fmicb.2020.01914

**Published:** 2020-08-28

**Authors:** Sara Hernando-Amado, Teresa M. Coque, Fernando Baquero, José L. Martínez

**Affiliations:** ^1^Centro Nacional de Biotecnología, Consejo Superior de Investigaciones Científicas (CSIC), Madrid, Spain; ^2^Hospital Universitario Ramón y Cajal, Instituto Ramón y Cajal de Investigación Sanitaria (IRYCIS) and Centro de Investigación Biomédica en Red Epidemiología y Salud Pública (CIBERESP), Madrid, Spain

**Keywords:** One Health, Global Health, antibiotic resistance, waste water, farming

## Abstract

Antibiotic resistance is a problem for human health, and consequently, its study had been traditionally focused toward its impact for the success of treating human infections in individual patients (individual health). Nevertheless, antibiotic-resistant bacteria and antibiotic resistance genes are not confined only to the infected patients. It is now generally accepted that the problem goes beyond humans, hospitals, or long-term facility settings and that it should be considered simultaneously in human-connected animals, farms, food, water, and natural ecosystems. In this regard, the health of humans, animals, and local antibiotic-resistance–polluted environments should influence the health of the whole interconnected local ecosystem (One Health). In addition, antibiotic resistance is also a global problem; any resistant microorganism (and its antibiotic resistance genes) could be distributed worldwide. Consequently, antibiotic resistance is a pandemic that requires Global Health solutions. Social norms, imposing individual and group behavior that favor global human health and in accordance with the increasingly collective awareness of the lack of human alienation from nature, will positively influence these solutions. In this regard, the problem of antibiotic resistance should be understood within the framework of socioeconomic and ecological efforts to ensure the sustainability of human development and the associated human–natural ecosystem interactions.

## Introduction

The problem of antibiotic resistance (AR) has been traditionally addressed by focusing on human-linked environments, typically health care facilities. Nevertheless, it is now generally accepted that most ecosystems may contribute to the selection and spread of AR (Aminov, 2009; Martinez et al., 2009; Davies and Davies, 2010; Martinez, 2014; Berendonk et al., 2015; Larsson et al., 2018). A key conceptual point is that, based on cultural, humanitarian, and economic reasons, we have historically preserved the health of individual humans and farming animals. To that purpose, the same families of antimicrobial agents have been used. As a consequence, their positive (healing) and negative (selection of AR, therapeutic failure) effects have influenced the common health of humans and animals in particular locations (One Health). The concept *One Health*, first used in early twentieth century, expands the integrative thinking about human and animal medicine, including for the first time ecology, public health, and societal aspects (Zinsstag et al., 2011). In the case of AR, the One Health perspective focuses on the risk assessment of emergence, transmission, and maintenance of AR at the interface between humans, animals, and any other linked (local) environment (Robinson et al., 2016; Jean, 2017). Consequently, the application of One Health approaches demands integrative surveillance tools and interventions based on multidisciplinary approaches that include ecological and sociodemographic factors, besides more classic epidemiological models.

Global Health is based on a broad collaborative and transnational approach to establish “health for all humans.” In this case, it focuses AR at a general (global) scale, considering that the selection and global spread of antibiotic-resistant bacteria (ARBs) and antibiotic resistance genes (ARGs) are a problem that influences the health of human societies with disparate social and economic structures and is linked to many societal and ecological factors (Chokshi et al., 2019). Interventions to reduce AR burden in a global world certainly require common and integrated policy responses of countries, international organizations, and other actors (stakeholders included). Its goal is the equitable access to health and minimizing health risks all over the globe. Besides its objective aspects (i.e., how travelers, migrating birds, or international commerce may contribute to AR spread), it has important international political aspects. It focuses in how countries and international organizations address the elements connecting and potentially spreading AR among humans, animals, and natural ecosystems at the Earth scale (Wernli et al., 2017). In summary, the problems and the potential solutions concerning AR are not confined to particular regions, but have a global dimension: a problem for all humans, animals, and natural ecosystems, which should be solved with interventions aiming to improve health for all of them (Brown et al., 2006; Koplan et al., 2009; Laxminarayan et al., 2013). In the context of AR, a healthy environment would be an environment where AR is low or can be controlled by human interventions (Hernando-Amado et al., 2019; Andersson et al., 2020).

Of course, the Global Health concept of “health of an environment” (Iavarone and Pasetto, 2018; Pérez and Pierce Wise, 2018; Bind, 2019; van Bruggen et al., 2019) or, in general, Planetary Health (Lerner and Berg, 2017), has an unavoidable anthropogenic flavor. In practice, we consider “healthy environments” or “healthy ecosystems” those that minimize their current or their potential harm for the human individual or the society, in our case for AR. In other words, we adopt a selfish strategy, which should be necessarily implemented by the international (global) institutions. Selfishness (Kangas, 1997) applies mainly to individuals, but also to societal groups. However, these groups have not enough possibilities to act alone in the case of infectious diseases in general and AR in particular, which may expand worldwide. Therefore, individual selfishness for health should be integrated in local One Health and also in Global Health actions. The goal of controlling AR is a highly complex one, and its dimension has been compared to climate change or biodiversity loss, problems where individual actions are not enough for providing a solution, and consequently, individual freedom is confronted with collective responsibility (Looker and Hallett, 2006).

The construction of human societies reflects the tension between individual freedom and social rules/laws. The implementation of different social rules/laws for regulating human activities within a society is mainly based on moral (as Kant’s categorical imperative (Kant, 1785) or religious-based brotherhood (Matthew 22:35–40) statements), social stability (as anticrime laws; Schiavone, 2012), organizative (type of government and how it is formed, group identity), and efficacy (as antitrust laws; Ricardo, 1821) arguments. However, these arguments mainly apply for establishing the socioeconomic organization as well as the individual welfare within a society. The situation concerning human health is somehow different. There are individual diseases, such as cancer or stroke, and social diseases, such as transmissible infections. For the firsts, social norms (as consciousness of the importance of the control of cholesterol, excess sugar uptake, or hypertension levels) are well established, and even laws (non-smoking regulations) had been implemented in occasions. However, the main impact of these regulations is at the individual health level (Wikler, 2002), because the associated diseases are not physically transmissible. A different situation happens in the case of infectious diseases in general and of AR in particular. For these diseases, everything that happens in a single person affects any one around. Further, the fact that an ARG emerging in a given geographic area can spread worldwide implies that neither individual norms nor country-based norms have been sufficient until now to counteract the worldwide spread of AR.

**FIGURE 1:**
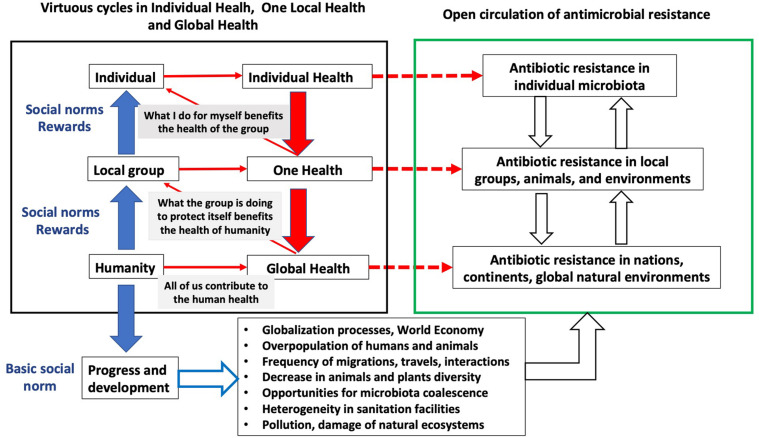
How the interactions among individual health, One Health, Global Health, and social norms influences antibiotic resistance. The right panel shows the different levels of dissemination of antibiotic resistance. In the left panel, the different types of norms (from individual to global norms) that can impact antibiotic resistance at each level are shown. These norms influence all levels of transmission: the individual promotes (red arrows) his own individual health, but doing it also promotes the health of the group, and the health of the group promotes Global Health of the human society at large. At each level, there is a positive action (red broken lines) on antibiotic resistance. Such dynamics largely depends on social norms (blue arrows) rewarding the individual or the groups whose behavior promotes health. Below the left panel, the basic social norm, progress and development, has consequences on the whole ecobiology of the planet (lower panel with bullet points), influencing the undesirable open circulation of antimicrobial resistant bacteria (with their mobile genetic elements) and antibiotic resistance genes.

One important aspect of laws in democratic societies is that they must be well accepted by the community, so that the acceptation of social norms usually comes first than their implementations as rules/laws. Actually, the efficiency of democracy for responding to social crisis (as current AR or COVID-19 crises), in opposition to other more autocratic regimens where decisions are implemented top-down, had been the subject of debate from the early beginning of democratic revolutions (Tocqueville, 1838; Hobbes, 1968; Rousseau, 1974; Spinoza, 2007). In this regard, it is important to remark that One Health aspects of AR can be tackled in the basis of country-level regulations that are linked to the socioeconomic and cultural aspects of each country (Chandler, 2019; Chokshi et al., 2019). However, because global Earth governance does not exist, Global Health control of AR is based on recommendations, rather than in rules/laws. Consequently, the acceptance of social norms, starting within individuals or small organizations and expanding throughout the whole society (Figure 1), is fundamental to provide global solutions to the AR problem (Nyborg et al., 2016; Chandler, 2019). The acceptance by the community of these social norms, considering that the way of promoting these norms might differ in different parts of the world (Cislaghi and Heise, 2018; Cislaghi and Heise, 2019), largely depends on the transfer to the society of the knowledge required to understand the mechanisms and the impact for human health of the emergence and transmission of AR, an information that is discussed below.

## Defining the Bricks Building Up Antibiotic Resistance in a Globalized World

The classic definition of AR is based only on the clinical outcome of the infected patient. An organism is considered resistant when the chances for the successful treatment of the infection it produces are low (Martinez et al., 2015). This definition, which is the most relevant in clinical settings, presents some limitations for studies based on One Health approaches that include the analysis of non-infective organisms, which lack a clinical definition of resistance, as well as analysis of the distribution of ARGs, in several occasions, using non–culture-based methods (Martinez et al., 2015). Even in the case of animal medicine, antibiotic concentration breakpoints defining resistance are still absent for some veterinary-specific antimicrobials and poorly defined for different types of animals with disparate weights, which would influence the availability of the drug inside animal body (Toutain et al., 2017; Sweeney et al., 2018). To analyze AR beyond clinical settings, the term *resistome*, understood as the set of genetic elements that can confer AR, irrespectively of the level of resistance achieved, in a given organism/microbiome was coined (D’Costa et al., 2006; Wright, 2007; Perry et al., 2014).

AR acquisition is the consequence of either mutation (or recombination) or recruitment of ARGs through horizontal gene transfer (HGT), transformation included. AR mutations are generally confined to their original genomes, propagating vertically and not spreading among bacterial populations, although some few exceptions of horizontal transfer of chromosomal regions containing AR mutations have been described (Coffey et al., 1991; Ferrandiz et al., 2000; Novais et al., 2016; Nichol et al., 2019). The set of mutations that confer AR can be dubbed as the mutational resistome. Current whole-genome-sequencing methods of analysis can allow defining the mutational resistome in an isolated microorganism (Cabot et al., 2016; Lopez-Causape et al., 2017). However, they are not robust enough yet for determining the mutational resistome in metagenomes. Consequently, the impact of these analyses in One Health studies is still limited and will not be further discussed in the present review.

Concerning their relevance for acquiring AR, ARGs can be divided in two categories. The first one comprises the genes forming the intrinsic resistome (Fajardo et al., 2008), which includes those that are naturally present in the chromosomes of all (or most) members of a given bacterial species and have not been acquired recently as the consequence of antibiotic selective pressure. Despite that these genes contribute to AR of bacterial pathogens, they are responsible just for the basal level of AR, which is taken into consideration when antibiotics are developed. In this regard, unless these genes, or the elements regulating their expression mutate, they are not a risk for acquiring resistance and have been considered as phylogenetic markers (Martinez et al., 2015). Further, it has been discussed that these genes may contribute to the resilience of microbiomes to antibiotic injury (Ruppe et al., 2017b), hence constituting stabilizing element of microbial populations when confronted with antibiotics more than a risk for AR acquisition by pathogens.

The second category, dubbed as the *mobilome*, is formed by ARGs located in mobile genetic elements (MGEs) that can be transferred both vertically and horizontally, hence allowing AR dissemination among different bacteria (Frost et al., 2005; Siefert, 2009; Jorgensen et al., 2014; Lange et al., 2016; Martinez et al., 2017).

While the analysis of the resistome of microbiota from different ecosystems has shown that ARGs are ubiquitously present in any studied habitat (D’Costa et al., 2006; Walsh, 2013; Jana et al., 2017; Lanza et al., 2018; Chen et al., 2019b), the impact of each one of these ARGs for human health is different. Indeed, it has been stated that the general resistome of a microbiome is linked to phylogeny and to biogeography, indicating that most ARGs are intrinsic and do not move among bacteria (Pehrsson et al., 2016). However, some ARGs escape to this rule and are shared by different ecosystems and organisms (Forsberg et al., 2012; Fondi et al., 2016). These mobile ARGs, frequently present in plasmids (Tamminen et al., 2012; Pehrsson et al., 2016), are the ones that are of special concern for human health.

Although not belonging to the antibiotic resistome, genes frequently associated with resistance to other antimicrobials, such as heavy metals or biocides, as well as the genes of the MGEs backbones, eventually involved in the transmission and selection of ARGs among microbial populations, the mobilome at large, are also relevant to track the emergence and dissemination of AR among different habitats (Lanza et al., 2015; Martinez et al., 2017; Baquero et al., 2019).

HGT processes are recognized as the main mechanisms for transmission of genetic information (Baquero, 2017). From the ecological point of view, HGT should be understood as a cooperative mechanism that allows the exploitation of common goods as ARGs (Baquero et al., 2019) by different members within bacterial communities. In fact, some studies suggest that the ecological consequences of HGT events in AR evolution are contingent on the cooperation of complex bacterial communities, besides the acquisition of individual adaptive traits (Smillie et al., 2011). However, the understanding of the ecological causes and consequences of ARGs transmission among organisms and microbiomes is still limited from the One Health and Global Health perspectives.

**FIGURE 2:**
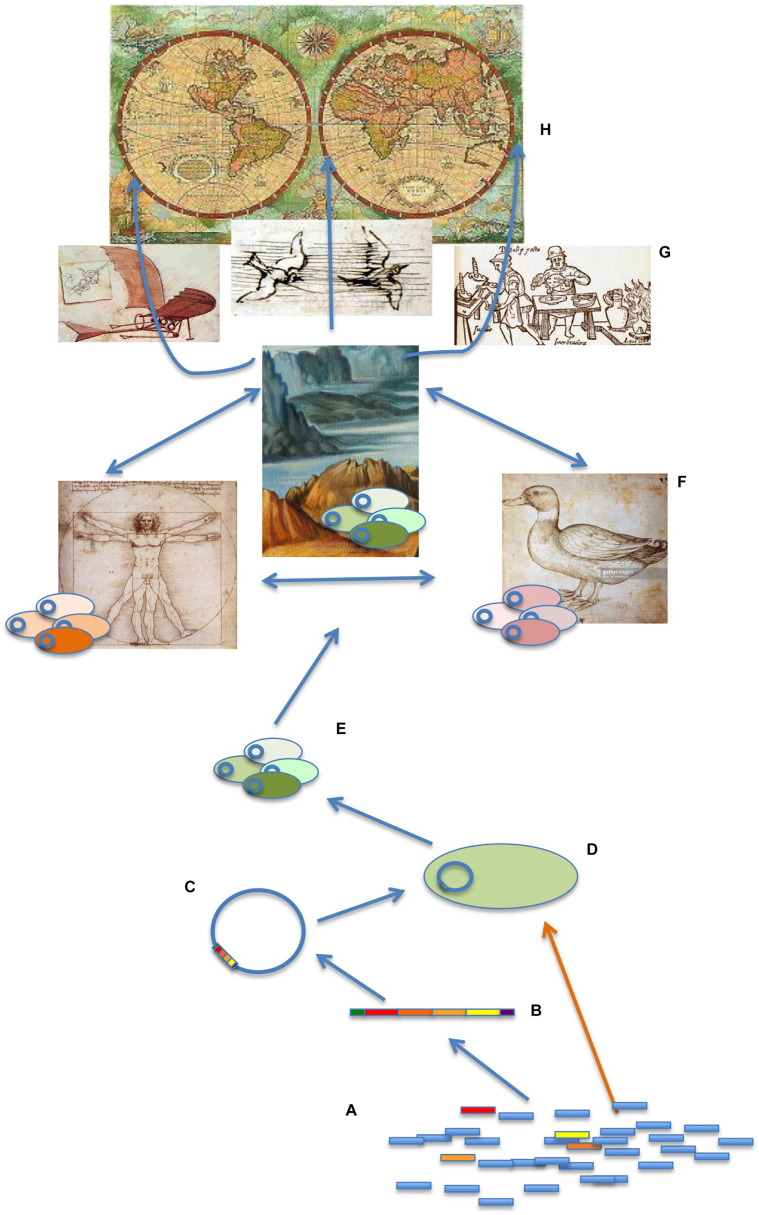
Genetic, ecological, and socioeconomic elements mediating the transmission of antibiotic resistance. ARGs are ubiquitously present in any studied microbiome **(A)**. However, only a few of them are transferred to human/animal pathogens, hence constituting a health problem. The genetics events implied include the acquisition of ARGs by gene-recruiting genetic elements such as integrons **(B)**; the integration of these elements in MGEs as plasmids, bacteriophages, or insertion conjugative elements **(C)**; and the acquisition of these elements by specific bacterial clones **(D)**. These ARBs can share these elements among the members of gene-sharing communities **(E)** and also move among different ecosystems, including humans, animals (particularly relevant farm animals), and natural ecosystems (with a particular relevance for water bodies). The connection of these ecosystems, as well as the reduced diversity of animals, plants, and in general habitats as the consequence of human activities, allows the different microbiomes to be in contact, favoring ARGs transmission among the microorganism they encompass **(F)**. This transmission is facilitated at the global scale by travel, animal migration, trade of goods, and eventually by meteorological phenomena, climate change included **(G)**, hence producing a Global Health problem **(H)**. While most studies on the dissemination of ARGs focus on MGEs (Davies, 1997; Muniesa et al., 2013; Lanza et al., 2015; Garcia-Aljaro et al., 2017), recent works suggest that the contribution of natural transformation (orange arrow), allowing the direct uptake of ARGs by natural competent microorganisms, may have been underestimated (Domingues et al., 2012; Blokesch, 2017). Further, competence can occur due to interbacterial predation (Veening and Blokesch, 2017), a biological interaction that may facilitate the acquisition of beneficial adaptive traits by predator bacterial species (Cooper et al., 2017; Veening and Blokesch, 2017). Other HGT mechanisms, such as DNA packing in extracellular vesicles (ECV) or transference of DNA through intercellular nanotubes, also seem to be relevant in nature (Dubey and Ben-Yehuda, 2011; Fulsundar et al., 2014). While the biotic conditions that may enhance HGT have been studied in detail, less is known concerning abiotic modulation of ARGs transfer. Under contemporary conditions, at least 10^24^ microorganisms are affected by a freeze-and-thaw cycle, at least 10^19^ are agitated by sand, and at least 10^17^ are subjected to conditions suitable for electrotransformation every year (Kotnik and Weaver, 2016).

HGT-mediated AR is a hierarchical process (Figure 2) in which ARGs are recruited by gene-capture systems as integrons and afterward integrated in MGEs as plasmids, insertion conjugative elements, or bacteriophages (Frost et al., 2005; Garcia-Aljaro et al., 2017; Gillings et al., 2017; Botelho and Schulenburg, 2020), which afterward are acquired by specific bacterial clones. Selection at each of these levels will also select for all the elements involved in AR spread. For instance, the acquisition of an ARG by a clone may promote the expansion of the latter (and of all the genetic elements it contains, other ARGs included) in antibiotic-rich environments, such as hospitals or farms (Martinez and Baquero, 2002; Schaufler et al., 2016), and *vice versa*, the introduction of an ARG in an already successful clone may increase the chances of this resistance gene for its dissemination even in environments without antibiotics, unless the associated fitness costs are high. In this sense, if ARG acquisition reduces the fitness, and this implies a decreased capability for infecting humans (see below), the burden for human health might eventually be lower. Nevertheless, it is relevant to highlight that AR transmission cannot be understood just by analyzing the genetic mechanisms involved and the consequences of such acquisition for the bacterial physiology. Indeed, as discussed below, there are ecological and socioeconomic elements that strongly influence AR dissemination.

## Highway to Antibiotic Resistance

The evolution of AR comprises the emergence, the transmission, and the persistence of ARBs (Martinez et al., 2007; Baquero et al., 2011). Concerning human health, selection of ARBs/ARGs is particularly relevant at the individual health level, whereas transmission is a main element to be taken into consideration at the One Health and Global Health levels (Figure 2). Indeed, unless AR is transmitted, it will be just an individual problem that would not affect the community at large.

It is generally accepted that non-clinical ecosystems are often primary sources of ARGs (Davies, 1994). As above stated, after their capture and integration in MGEs (Figure 2), ARGs and their bacterial hosts can contaminate different ecosystems, which might then be involved in their global spread (Martinez, 2012; Fondi et al., 2016; Gillings, 2017; Gillings et al., 2017). This means that nearly any ecosystem on Earth, along with the human-driven changes produced in it, may modulate evolution of AR. Importantly, the huge escalation and worldwide expansion of a limited set of animals, plants, and their derived products, including foods, due to the anthropogenic selection of a few breeds and cultivars for mass production in livestock and agricultural industries (Okeke and Edelman, 2001; Zhu et al., 2017) of economic interest have collapsed the variability and biodiversity of animals and plants (Seddon et al., 2016). Because these organisms harbor particular host-adapted bacteria, which are frequently under antibiotic challenge, this situation, together with the ecological similarities of human habitats, might favor AR spread (Martiny et al., 2006; Manyi-Loh et al., 2018). Indeed, while in underdeveloped areas of the world food animals are very diverse, intensive farming, common in developed countries, ensures a “shared-stable” environment where only the most productive types prevail (Kim et al., 2017). The common genetic origin of these types and the process of microbiota acquisition from nearby animals in intensive farming should homogenize also their microbiomes with consequences for AR dissemination. Actually, it has been shown that the loss of microbial diversity may favor AR spread (Chen et al., 2019a). Note that, beyond the transmission of particular AR spreading clones, AR is expected to spread in farms by the modification (eventually homogenization) of animals’ microbiota. Notwithstanding, even farm workers are subject to microbiome acquisition from animals, leading to microbiome coalescence (Baquero et al., 2019; Sun et al., 2020). It is to be noticed, and the recent COVID-19 crisis exemplifies it, that besides economic development, cultural habits are relevant in the use of animals for food, a feature that has not been analyzed in detail, particularly with respect to their role as vectors potentially involved in AR dissemination.

**FIGURE 3:**
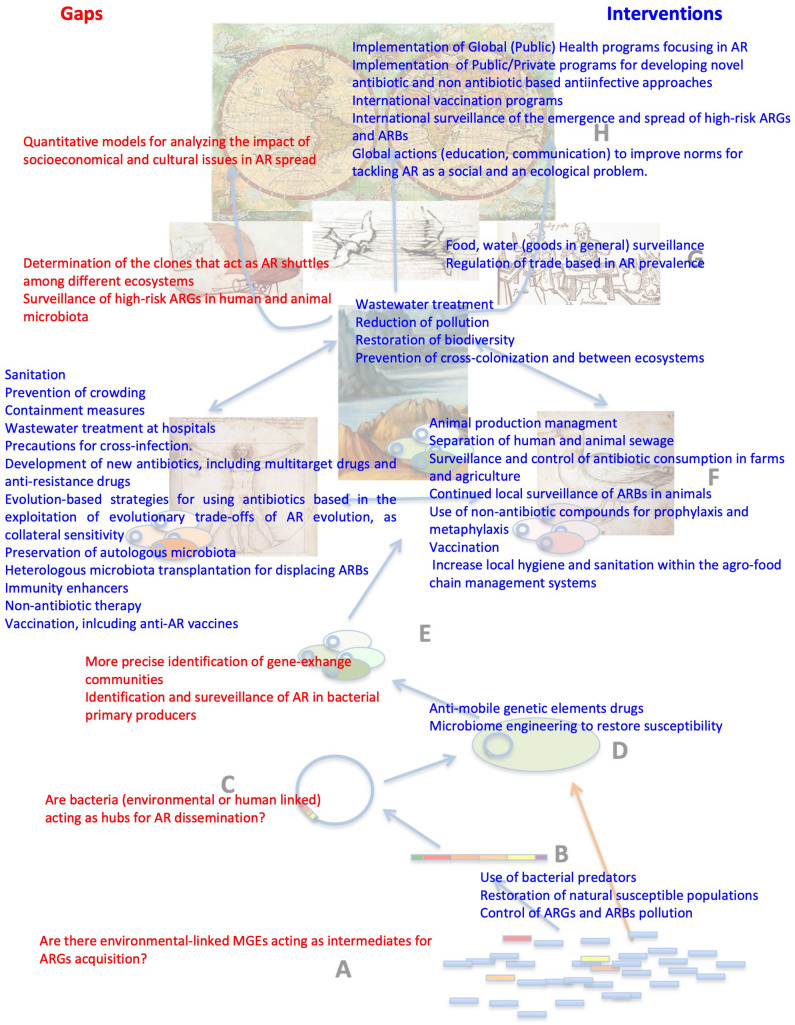
Local and global intervention strategies to tackle AR and knowledge gaps that could help improve existing ones. Most interventions for reducing antibiotic resistance are based on impairing the selection of ARBs/ARGs, which is just the first event in AR spread. Our main goal, as for any other infectious disease, would be reducing transmission. This does not mean that selective pressure is not relevant for transmission. Indeed, without positive selection, HGT events are not fixed, allowing the enrichment of some ARGs that are consequently more prone to diversification, both because they are more abundant and more frequently subjected to selection (Davies, 1997; Martinez, 2009a, b; Salverda et al., 2010) and because they can explore different landscapes when present as merodiploids in multicopy plasmids (Rodriguez-Beltran et al., 2018). Therefore, reducing the selective pressure, either due to antibiotics or by other coselecting agents as heavy metals, still stands as a major intervention against AR emergence and transmission. To address this issue, we need to know more on the amount of pollutants, their selective concentrations, and their mechanisms of coselection and cross-selection in different ecosystems. This is a general example illustrating the gaps in knowledge in the AR field that need to be filled as well as strategies that may help in tackling this problem. The figure includes several other examples of the gaps of knowledge (red) that require further studies and the interventions (blue) that may help to tackle AR.

Despite that the homogenization of hosts may help in AR transmission, the spread of ARBs has some constraints, because the differential capability of each bacterial clone for colonizing different hosts may modulate their dissemination. Indeed, while some species and clones are able to colonize/infect different animal species, humankind included, several others present some degree of host specificity (Price et al., 2017; Sheppard et al., 2018). Further, it has been shown that the capacity to colonize a new host is frequently associated with a reduction in the capacity for colonizing the former one. The same happens for mobile ARGs; they are encoded in MGEs that present different degrees of host specificity, which defines the formation of gene-exchange communities, where the interchange of genetic material among members is facilitated (Skippington and Ragan, 2011). Conversely, the incorporation of different replicons and modules within plasmid backbones, a feature increasingly reported (Douarre et al., 2020), would enable ARG replication in different clonal/species background and thus modify the community network of ARGs. Actually, the risk for humans of animal-based AR seems to be linked in most cases to shuttle, generalist clones able to colonize humans and particular animals (Price et al., 2017; Sheppard et al., 2018). The understanding of the elements driving the transfer of AR among animals, humans included (Figure 3), requires the comprehensive survey of the clones and ARGs that are moving among them (European Food Safety Authority et al., 2020). Tools to track the global epidemiology of antimicrobial-resistant microorganisms such as Bigsdb (Jolley et al., 2018) or comprehensive databases of ARGs, ideally providing information of their mobility (Zankari et al., 2012; Alcock et al., 2020), are fundamental for studying AR transmission at a global level.

It is worth mentioning that, because humans constitute a single biological species, the human-associated organisms spread easily among all individuals. In fact, more prominent differences in humans’ microbiome composition can be observed between individuals than among ethnic groups, even though, as expected, the resemblance in microbiotas is higher among those groups that are geographically clustered (Deschasaux et al., 2018; Gaulke and Sharpton, 2018). Some groups of human population are, however, more prone to acquire ARBs, due either to socioeconomic or to cultural factors. In LMICs (low- to medium-income countries) and BRICS (Brazil, Russia, India, China, and South Africa) countries, the combination of wide access to antibiotics, weak health care structures, and poor sanitation defines certainly a dangerous landscape. Moreover, the progressive aging of the Western population might favor the establishment and further expansion of an elderly reservoir of ARBs and ARGs, an issue that deserves further studies. The hypothesis that the microbiome of elder people might be a reservoir of AR is based not only on their cumulative history of antibiotic exposure and contacts with health care centers, but also on the rampant use of antibiotics of this population more prone to suffer from acute, chronic, or recurrent infections. Significant worldwide advances in the organization of medical care of the elderly people lead to frequent hospitalizations, but health care centers may also facilitate the selection and further amplification of AR in the community. In addition, this may subsequently favor the entry of high-risk clones and of ARGs in the hospital setting (Hujer et al., 2004).

As stated above, there is a global increasing permeability of the natural biological barriers that have historically prevented bacterial dissemination through different ecosystems. Besides local spread of AR in environments shared by animals and humans, which has to be addressed under a One Health approach, AR can disseminate worldwide (Figure 2) by economic corridors that promote the global interchange of goods and trade or human travelers or by natural bridges, such as animal migration paths or natural phenomena such as air and water movements (Okeke and Edelman, 2001; Baquero et al., 2008; Allen et al., 2010; Overdevest et al., 2011; Kluytmans et al., 2013; Fondi et al., 2016). The result is the appearance of similar ARBs and ARGs in different geographic areas. As the consequence, AR is a Global Health problem in the sense that an ARB that emerges in a given place can rapidly spread worldwide. Indeed, multidrug-resistant bacteria, similar to those encountered in clinical settings, have been detected in human isolated populations that were not previously in contact with antibiotic, as well as in wildlife (Clemente et al., 2015). This indicates that pollution with ARGs is present even in places where antibiotic concentrations are low (Kümmerer, 2004) and might involve mechanisms of transmission that do not require selection. For instance, migrating birds can carry enteropathogenic bacteria resistant to different antibiotics (Middleton and Ambrose, 2005; Poeta et al., 2008), and international travelers, even those not receiving antibiotic treatments, also contribute to AR transfer among different geographic regions (Murray et al., 1990; Reuland et al., 2016). In the group of long travelers are refugee people, in which dissemination of multidrug-resistant strains is favored by the poor sanitary conditions and overcrowding camps that refugees confront (Maltezou, 2016).

A final issue concerning AR is its stability in the absence of selection. It has been proposed that the acquisition of AR reduces bacterial competitiveness in the absence of antibiotics (fitness costs) (Andersson and Hughes, 2010; Martinez et al., 2011); certainly, a wishful proposition such as, if true, the reduction in the use of drugs or eventually antibiotic-cycling strategies should decrease AR (Beardmore et al., 2017). Nevertheless, eliminating the use of an antibiotic does not produce a full decline of AR (Sundqvist et al., 2010). In fact, different studies have shown that AR not always reduces fitness but also can even increase bacterial competitiveness (Andersson and Hughes, 2010; Schaufler et al., 2016). In addition, compensatory mutations or physiological changes that restore fitness can be selected in resistant bacteria (Andersson, 2006; Schulz zur Wiesch et al., 2010; Olivares et al., 2014). It is a fact, however, that although ARBs are found nearly everywhere, including wild animals, natural ecosystems, or people from isolated populations without contact with antibiotics, among others (Durso et al., 2012; Clemente et al., 2015; Alonso et al., 2016; Fitzpatrick and Walsh, 2016; Power et al., 2016), AR prevalence is consistently lower when antibiotics are absent, which suggests that pollution may impact AR, a feature that is discussed below.

## Antibiotic Resistance in an Anthropogenically Impacted World

Pollution of natural ecosystems is associated with activities that have driven relevant economic transition, in principle favoring human welfare, such as mining, industry, intensive land use, or intensive farming, among others. Notwithstanding, globalization of health services, as well as the shift toward intensive farming, besides their positive contribution to human well-being, has rendered an increasing pollution by compounds with pharmacological properties of natural ecosystems, particularly water bodies, which may disrupt the stability of these ecosystems (Oldenkamp et al., 2019). Among them, antibiotics are considered the most relevant cause of AR selection. Despite regulations for reducing their use (Van Boeckel et al., 2017), a substantial increase in global antibiotic consumption has occurred in the last years, and an even greater increase is forecasted in the next years (Klein et al., 2018).

However, antibiotics are not the unique pollutants that can prime the selection and spread of AR. In this regard, it is important to highlight that heavy metals are one of the most abundant pollutants worldwide (Panagos et al., 2013). Their abundance results from anthropogenic-related activities, such as mining, industry, agriculture, farming, or aquaculture and even for therapeutic use in ancient times. Importantly, they may persist in nature for long periods of time. Further, likely because metal pollution occurred before the use of antibiotics, heavy metal resistance genes were incorporated to MGE backbones before ARGs (Mindlin et al., 2005; Staehlin et al., 2016). This means that heavy metals may coselect for MGEs and the ARGs they harbor (Partridge and Hall, 2004; Staehlin et al., 2016; Zhao et al., 2019a). Even more, the presence of heavy metals, as well as of biocides or sublethal antibiotic concentrations (Jutkina et al., 2018; Zhang et al., 2018), may stimulate HGT, as well as modify the dynamics of antibiotics, such as tetracyclines, in natural ecosystems (Hsu et al., 2018). Coselection may also occur when a single resistance mechanism, such as an efflux pump, confers resistance to both heavy metals and antibiotics (cross-resistance) (Pal et al., 2017).

Although most published works analyze the effect of different pollutants on their capacity to select ARBs or ARGs, it is important to highlight that ARGs should also be considered pollutants themselves. Actually, a recent work indicates a close relationship between the abundance of ARGs and fecal pollution (Karkman et al., 2019). In this respect, it is worth mentioning that, differing to classic pollutants, ARGs/ARBs are not expected to disappear along time and space, but rather, their abundance may even increase as the consequence of selection and transmission (Martinez, 2012). While the direct selection of AR by antibiotics or the coselection mediated by other pollutants, as the aforementioned heavy metals, has been discussed (Wales and Davies, 2015), the effect of other types of human interventions on the dissemination of ARGs and ARBs through natural ecosystems has been analyzed in less detail. As an example, it has been proposed that wastewater treatment plants, where commensals, ARBs, ARGs, and antibiotics coexist, could act as bioreactors favoring the selection and transmission of ARGs between different organisms (Rizzo et al., 2013; Su et al., 2017; Manaia et al., 2018), although evidences supporting this statement are scarce (Munck et al., 2015; Azuma et al., 2019).

In addition to the aforementioned pollutants with a direct effect in AR selection, it is worth noting that there are other abundant contaminants, such as sepiolite (present in cat litters or used as a dietary coadjuvant in animal feed) or microplastics, present in almost all aquatic ecosystems, which can favor the transmission of ARGs or MGEs between bacterial species (Rodriguez-Beltran et al., 2013; Kotnik and Weaver, 2016; Arias-Andres et al., 2018), hence amplifying the AR problem at a global scale.

Finally, the possible effect of climate change on the spread of AR is worth mentioning. Indeed, it modifies the biogeography of vectors (such as flies, fleas or birds) involved in the spread of infectious diseases (Fuller et al., 2012; Beugnet and Chalvet-Monfray, 2013). In addition, the increase of local temperatures seems to correlate with an increased AR abundance in common pathogens (MacFadden et al., 2018). Besides, climate change is affecting ocean currents (Martinez-Urtaza et al., 2016), which may allow the intercontinental distribution of ARBs and ARGs (Martinez, 2009a, b). Although this phenomenon might contribute to the globalization of AR, further research is needed to clearly demonstrate a cause–effect relationship.

It is relevant to mention that increased pollution and climate change are the unwanted consequences of human development. It would then be worth discussing how human development in general may impact (positively and negatively) AR, a feature that is analyzed below.

## Antibiotic Resistance as a Global Development Problem

Human development is a necessity of our human behavior, although different models of development have been and are proposed, each one producing different impacts in the structure of human societies and on the preservation and stability of natural ecosystems (Fenech et al., 2003; Farley and Voinov, 2016; Seddon et al., 2016). Nevertheless, even for different socioeconomic models, there are some social norms that tend to be widely accepted, in particular those aiming to improve individual well-being. This implies the establishment of a society of welfare, understood as a right of any human on Earth, a feature that depends on the economic development, and can be particularly relevant in the case of transmissible infectious diseases in general and of AR in particular.

A continuously repeated mantra in worldwide AR policies is that the abusive consumption of antibiotics for the treatment or prevention of infections in humans and animals constitutes the major driver of AR. However, we should keep in mind that antibiotics constitute an important example of human progress supporting individual and global human health. In fact, the origin of the massive production of antimicrobials was a consequence of the needs resulting from World War II in the 1940s. This was followed by many decades of human progress, most importantly by the common understanding of equal human rights, which was followed by the economic and social development (including medicine and food industry) of densely populated regions in the planet, including India and China. These countries are currently among the leaders in the production and consumption of antimicrobial agents. Notwithstanding, as in any area of economy, progress bears a cost that, in this case, is antibiotic pollution of the environment, globally accelerating the process of the emergence, the transmission, and the persistence of ARBs (Martinez et al., 2007; Baquero et al., 2011).

The non-controlled use of antibiotics is facilitated in LMICs with disparate economic growth by different factors. Heterogeneous regulation of antibiotic sales and prescriptions (often weak or missing) and the increase of online on-bulk sales in recent years contribute to their overuse (Mainous et al., 2009). Most of live-saving medicines represent out-of-pocket costs in most LMICs, which led to an exacerbated use of cheap (usually old and less effective) antibiotics, phasing out their efficacy and increasing the demands and prices for the most expensive ones, eventually resulting in treatment unavailability (Newton et al., 2011). Further, the cost of treating AR infections is much higher than that of treating susceptible ones, which is increasing the cost of health services (Wozniak et al., 2019). Conversely, the growing economic capability of LMICs in the BRICS category triggers the access of the population to health services and last-resort antibiotics. These countries also face a sudden high demand for meat and thus a prompt industrialization of animal production that has favored the misuse of antibiotics for growth promotion facilitated by their online availability (Mainous et al., 2009). In addition, counterfeit or substandard antibiotics recently become a serious global problem (Gostin et al., 2013), which is exacerbated in LMICs, where they represent up to a third of the available drugs. Noteworthy, 42% of all reports received by the WHO Global Surveillance and Monitoring System on substandard and falsified medicines worldwide come from Africa, and most of them correspond to antimalarials and antibiotics (Newton et al., 2011; Gostin et al., 2013; Hamilton et al., 2016; Petersen et al., 2017).

Despite this situation, it is important to highlight that human consumption of antibiotics is an unavoidable need to preserve human health. In fact, most health problems dealing with infections in LMICs are still caused by a poor access to antibiotics, not by an excessive use of them. Proof of this is the fact that the distribution of antibiotics has reduced endemic illnesses and children mortality in Sub-Saharan Africa (Keenan et al., 2018). This means that, while a global decline in the use of antibiotics would be desirable to diminish the problem of AR, there are still several parts in the globe where antibiotic use should still increase to correctly fight infections. In fact, our primary goal should not be to reduce the use of antibiotics, but to ensure the effective therapy of infectious diseases for the long term. This does not mean that AR is not a relevant problem in LMICs; it means that reducing antibiotic use is not enough to solve the problem. Indeed, the current high morbidity and mortality due to infectious diseases (malaria, tuberculosis, low respiratory infections, sepsis, and diarrhea) in LMICs will be worsened in the absence or low efficiency of therapeutic treatments. Further, AR has economic consequences. According to World Bank, 24.1 million people could fall into extreme poverty by 2050 because of AR, most of them from LMICs (Jonas and World Bank Group Team, 2017).

Consequently, besides a Global Health problem, AR has an important economic impact (Rudholm, 2002), hence constituting a Global Development Problem, endangering not only the achievements toward the Millennium Development Goals but also the Sustainable Development Goals (van der Heijden et al., 2019). World Bank estimates that AR could impact the gross domestic product from 1 to 3.8%, which is even higher than what is estimated for the climate change (Jonas and World Bank Group Team, 2017). These economic foresights are linked to the threads of increased poverty, food sustainability, Global Health deterioration (associated with both food safety and affordability to health care), and environment protection. All these issues are also impacted by the overuse and misuse of antibiotics, its lack of effectiveness, and the affordability to medicines and health care (van der Heijden et al., 2019).

When talking about reducing antibiotic consumption, it is important to remind that up to two-thirds of overall antibiotic usage is for animal husbandry (Done et al., 2015). Further, recent work states that the use of antibiotics in crops, particularly in LMICs, might have been largely underestimated (Taylor and Reeder, 2020). Despite that evidences on the presence of common ARGs distributed among animals and humans were published decades ago (Bager et al., 1997; Wegener et al., 1997; Aarestrup, 1998; Aarestrup et al., 1998), and although the use of antibiotics as growth promoters has been banned in different countries (Cox and Ricci, 2008), they are still allowed in many others (Mathew et al., 2007). Of relevance is the fast increase of antibiotic consumption for animal food production in China (23% in 2010) and other BRICS countries (Van Boeckel et al., 2015). As stated previously, in these countries, increased income has produced a fast increase in meat products demand, due to changes in diet of their population. In addition, the increasing international competitiveness in meat production of these countries has fostered the rampant development of their industrial farming. Together with the fact that legislation on antibiotics use remains weak, this situation increases the risk of emergence of AR linked to animal production. Nevertheless, the problem is not restricted only to LMICs, because antibiotics consumption rose as well in the high-income countries as the United States (13%) (Van Boeckel et al., 2015), where approximately 80% of the antimicrobials purchased in 2011 were applied in livestock production as non-therapeutic administration (Done et al., 2015). The development of intensive methods of fish production has also contributed to the rise in the use of antimicrobials and the selection of resistance determinants that can be shared among fish and human bacterial pathogens (Cabello et al., 2013).

Economic development has facilitated as well more global transport, waste disposal, and tourism, favoring AR spread within and between different geographical areas (Ruppe et al., 2017a; Ruppe and Chappuis, 2017). However, economic growth can also reduce the AR burden, especially when it enables the development of regulations and infrastructures that might reduce the risks of infection and AR spread. This is particularly relevant in the case of public health interventions on food, water, and sewage. Because AR pathogens are mainly introduced in natural ecosystems through the release of human/animal stools (Karkman et al., 2019), the best way of reducing this impact is through the use of wastewater treatment plants, which are still absent in several places worldwide. Indeed, it has been described that drinking water is a relevant vehicle for the spread of ARBs in different countries (Walsh et al., 2011; Fernando et al., 2016) and that raw wastewater irrigation used for urban agriculture may increase the abundance of mobile ARGs in the irrigated soil (Bougnom et al., 2020). Notably, the analysis of ARGs in wastewaters has shown that the prevalence of ARGs in the environment in each country might be linked to socioeconomic aspects mainly related to economic development, as general sanitation, particularly the availability of drinking and wastewater treatments, malnutrition, number of physicians and health workers, human overcrowding, or external debt grace period (Hendriksen et al., 2019). The field of AR has mainly focused in the mechanisms of selection; the main driver for the increased burden of AR would be then the use of antibiotics itself. However, these results indicate that transmission, even in the absence of direct human-to-human contact, might be, at least, equally relevant. In this situation, an important element to reduce the AR burden will be to break the transmission bridges among different ecosystems that could be reservoirs of ARGs.

Even when wastewater-treatment plants are available, the presence of ARBs in drinking, fresh, and coastal waters, as well as in sediments nearby industrial and urban discharges, has been described in several countries (Ma et al., 2017; Leonard et al., 2018). As in the case of fecal contamination markers, a reduction in the amount of ARGs to non-detectable levels would be extremely difficult even when advanced water treatment procedures are applied. A standard definition of polluting ARB/ARG markers, as well as their acceptable levels, is then needed. This would be required not only for potable water, but also for water reutilization, as well as for land application and release of sewage effluents, because in all cases the reused water/sewage may carry ARBs and ARGs, together with pollutants, such as antibiotics, metals, biocides, or microplastics, which, as above stated, may select for AR (Baquero et al., 2008; Moura et al., 2010; Yang et al., 2017; Zhu et al., 2017; Larsson et al., 2018; Imran et al., 2019; Wang et al., 2020) and may even induce HGT.

The examples discussed above justify that human health in general and AR in particular are closely interlinked with economic development (Sharma, 2018). Economic differences are also found at individual level, because there is a positive relationship between economic status and health (Tipper, 2010). In addition, social behavior might also impact AR, a feature discussed in the following section.

## Social Norms and Tipping Points in Antibiotic Resistance: A Socioecological Approach

Different socioeconomic factors can modulate the spread of infective bacteria in general and of AR in particular. Among them, the increasing crowding of humans and foodborne animal populations favors transmission at the local level (One Health), whereas trade of goods and human travel (Figure 2) favor worldwide transmission (Global Health) (Laxminarayan et al., 2013; Hernando-Amado et al., 2019).

Besides these global changes in social behavior, linked to economic development, more specific socioeconomic factors (income, education, life expectancy at birth, health care structure, governance quality), sociocultural aspects (inequalities, uncertainty avoidance, integration of individuals into primary groups, gender biases, cultural long-term orientation), and personality dimension highly influence antibiotic use and AR transmission (Gaygısız et al., 2017). For instance, although the governance quality seems to be the most important factor associated with a proper antibiotic use, Western countries with distinct national culture patterns show different levels of antibiotics consumption (Kenyon and Manoharan-Basil, 2020). A better understanding of human social responses facing ailments, especially epidemics and antibiotic use, requires then a more detailed analysis of the differences between collectivistic (individuals living integrated into primary groups) and individually long-term oriented societies (oriented to future individual rewards) (Hofstede, 2001; Gaygısız et al., 2017; Kenyon and Manoharan-Basil, 2020).

Consistent with the sociological elements of AR, many of the aspects influencing AR reviewed above depend on social norms (Figure 1). In the classic view of the psychoanalyst Erich Fromm presented in his book “Escape From Freedom” (Fromm, 1941), human individual behavior is oriented to avoid being excluded from a higher social group. Indeed, not following social common rules can be eventually considered as a mental disorder; a sociopathology. A social norm is defined as a predominant behavioral pattern within a group, supported by a shared understanding of acceptable actions and sustained through social interactions within that group (Nyborg et al., 2016). In democratic societies, laws usually derive from already accepted social norms; otherwise, they would be changed, and in that sense, the establishment of accepted social norms for fighting AR is a prerequisite to implement the global approaches, based on worldwide rules, which are required for tackling this relevant problem.

Interestingly, the AR problem is a bottom-up process, where small emergent changes (in some type of individual patients, in some groups, in some locations) cumulatively escalate to gain a global dimension. Frequently, that occurs by crossing tipping points, that is, points where the local AR incidence becomes significant enough to cause a larger, eventually Global, Health problem. Because of that, the implementation of solutions should be adapted to the control of critical tipping points in the small groups of individuals to disrupt the bottom-up processes. However, as AR spread can occur everywhere and at any time, global surveillance and mechanisms of control should be implemented to prevent a top-down process of global AR expansion.

Individual selfishness for AR is the cornerstone of social norms. This concept was coined and developed by one of us over a decade ago (Baquero, 2007). Let us imagine that each individual is aware that each consumption of an antibiotic increases the personal risk of himself/herself or for his/her closer relatives (frequently exchanging microorganisms) of dying because of an antibiotic-resistant infection. The situation is analogous to the consumption of cholesterol-rich or highly salted food, or drinks with excess of sugar, concerning individual health. However, in the case of AR, it requires the understanding of the impact of individual actions at the global level. In this respect, anti-AR social actions should resemble more antitobacco and even general pollution/ecological campaigns.

At the individual level, there is inertia that precludes changing habits, until a tipping point is crossed and health is compromised. The conclusions of studies mainly based on long-term cohort analysis, such as the Framingham program for the influence of diet or smoking on personal cardiovascular disease (Mahmood et al., 2014), have become social norms that are naturally imposed by the ensemble of individuals. This creates a kind of societal culture, leading to appropriate individual behaviors, in occasions without the need of specific laws (diet), in occasion favoring the implementation of such laws (antismoking). However, we lack similar studies on issues such as these dealing with personal–familiar risks that have successfully shifted social norms, driven by groups of individuals and based on the promotion of individual behaviors in the case of AR.

Despite that quantitative models on how individual antibiotic use mayimpact AR at the population level are still absent, it is worthmentioning that a reduced antibiotic consumption has also begun tooccur in a number of countries just as a result of a change inindividual behavior (Edgar et al., 2009), and some tools and indicators to address these changes have been suggested (Ploy et al., 2020). The “tragedy of the commons” metaphor, first proposed in the XIX century (Lloyd, 1833) and later on discussed in 1968 (Hardin, 1968), has been used for addressing the sociology of AR, by showing how individual selfishness promotes antibiotic use, increases resistance, and influences the health of the community by impairing antibiotic efficacy (Baquero and Campos, 2003; Foster and Grundmann, 2006). Ensuring the prestige of individuals that follow the social rules is needed to counteract the tragedy of the commons. Nevertheless, it is important noticing that the tension between individual freedom and social rules that is inherent to the construction of democratic societies (Tocqueville, 1838; Hobbes, 1968; Rousseau, 1974; Spinoza, 2007) also applies here. One example of this situation is vaccination, considered in the last century as one of the most important advances to fight infectious diseases and now being the focus of antivaccination campaigns (Megget, 2020), a movement that has been considered by the WHO as one of the top 10 Global Health threats of 2019^1^. It is commonly accepted that social norms are mainly created by learning and education, a rational path that promotes health (Chen and Fu, 2018). Also, the increasing activities of “personalized medicine,” including antibiotic stewardship, follow the same trend (Gould and Lawes, 2016). However, the antivaccination movement is an example of how the narrative, as well as the use of decentralized, social information channels such as the Internet search, blogs, and applications to facilitate communication such as Twitter, Facebook or WhatsApp, is of particular relevance in the construction of social norms, not necessarily based on scientific and rational grounds (Jacobson et al., 2020; Scott and Mars, 2020).

The impact of social norms goes beyond human societies as human activities alter natural ecosystems; consequently, humans cannot be aliens of nature. We should then shape a socioecological system, linking the individuals, the groups, and the entire society, as well as natural ecosystems, also potentially damaged by AR, in a common multilevel adaptive system based on social norms and policies at the individual, local (One Health), and global (Global Health) scale (Levin et al., 2013). The recent crisis of COVID-19 illustrates the influence of social norms in the individual behavior. Each one of the individuals, protecting himself/herself, also protects the others. A person not wearing on face mask is frowned upon, and on the contrary, somebody attaching to the rules increases reputation. The individual adopts the right behavior being influenced by the judgment. of others. In addition, different political regimes (democracy or autocracy), as well as their organization (centralized, federal), together with the capacity of the health services to support the norms and their efficacy to communicate the chosen policy to the citizenry, may shape the individual responses to social norms (Greer et al., 2020; Häyry, 2020; Kavanagh and Singh, 2020).

Notwithstanding, two reasons that have been proposed to explain the low prevalence of COVID-19 in Japan were related with social norms more than with biological issues. These reasons, which are not common to other countries, were the socially accepted use of face masks and the mandatory vaccination of all the population against tuberculosis, which might protect from SARS-CoV-2 infection (Iwasaki and Grubaugh, 2020), a feature that is still to be confirmed.

The loss of social prestige of individuals taking antibiotics without prescription, as well as the pharmacies delivering these drugs or do not respect environmental protection, or the overconsumption of antibiotics in hospitals or in farms, or even in certain countries, is progressively constituting a “social norm,” converted in rules able to reduce AR emergence and spread. Of course, family and school education, as well as governmental campaigns, including the use of social media (Grajales et al., 2014) reinforces such social norms, which could allow the support of the society for the implementation of different interventions, some of them described below.

## Controlling Resistance: Local and Global Interventions

Controlling resistance not only requires establishing local interventions, which could be relatively easily implemented, but would also require global interventions that every country should follow, despite their disparate regulatory systems. Local and global interventions are necessarily intertwined; for example, the use of a new drug to treat a single individual depends on regulations at the county level (One Health approach), but the worldwide prevalence and transmission of resistance to this drug, as well as the regulations of its use, should be established internationally (Global Health approach).

Three main interventions to tackle AR have been historically considered: first, reduction of the antibiotic selective pressure by decreasing antimicrobials use; second, reduction of transmission of ARBs using improved hygienic procedures that prevent spread; third, development of novel antimicrobials with limited capacity to select ARBs or the design of new treatment strategies based on use of non–antibiotic-based approaches or, more recently, on the exploitation of trade-offs associated with AR evolution (Imamovic and Sommer, 2013; Gonzales et al., 2015; Barbosa et al., 2018; Imamovic et al., 2018). These interventions have been basically limited to local initiatives, applied mainly to hospitals and, more recently, to farms. However, AR has emerged and spread globally, in bacteria from different environments, so the health and dynamics of the global microbiosphere could be affected by antibiotics. In a sense, AR is affecting the Planetary Health (Lerner and Berg, 2017), and the needed interventions for tackling this problem cannot be restricted to hospital settings (Figure 3).

The proposed reduction in the use of antibiotics (Blaskovich, 2018) must be compensated with alternative approaches for fighting infectious diseases. In this regard, strategies based on improving the capability of the immune system for counteracting infections (Levin et al., 2017; Traven and Naderer, 2019) or the use of non-antibiotic approaches to prevent them, such as vaccines (Jansen and Anderson, 2018), may help to reduce the burden of AR infections. Indeed, vaccination against *Haemophilus influenzae* and *Streptococcus pneumoniae* has been demonstrated to be an effective intervention for reducing AR (Jansen and Anderson, 2018). However, while vaccination has been extremely useful to prevent viral infections, it has been less promising in the case of bacterial ones. Recent approaches, including reverse vaccinology, may help in filling this gap (Delany et al., 2013; Ni et al., 2017). Moreover, vaccination should not be restricted to humans, because veterinary vaccination can also contribute to animal wealth and farm productivity (Francis, 2018). Besides, the use of vaccines in animal production reduces the use of antibiotics at farms/fisheries, hence reducing the selection pressure toward AR.

Other strategies to reduce antibiotic selective pressure include the use of bacteriophages (a revitalized strategy in recent years) (Viertel et al., 2014; Forti et al., 2018), not only in clinical settings, but also in natural ecosystems (Zhao et al., 2019b), as well as the use of biodegradable antibiotics (Chin et al., 2018) or adsorbents, able to reduce selective pressure on commensal microbiome (De Gunzburg et al., 2015, 2017). Besides reducing the chances of selecting ARBs, the use of antibiotics adsorbents may preserve the microbiomes, reducing the risks of infections (Chapman et al., 2016). Importantly, the procedures for removing antibiotics should not be limited to clinical settings, but their implementation in wastewater treatment plants would reduce selection of AR in non-clinical ecosystems (Tian et al., 2020).

Concerning the development of new antimicrobials (Hunter, 2020), while there is a basic economic issue related to the incentives to pharmaceutical companies (Sciarretta et al., 2016; Theuretzbacher et al., 2017), the focus is on the possibility of developing novel compounds with low capacity for selecting AR (Ling et al., 2015; Chin et al., 2018). For this purpose, multitarget (Li et al., 2014) or antiresistance drugs, such as membrane microdomain disassemblers (Garcia-Fernandez et al., 2017), are also promising. Furthermore, antimicrobial peptides, with a dual role as immunomodulators and antimicrobials, may also help fight infections (Hancock et al., 2016). In fact, some works have shown that ARB frequently present collateral sensitivity to antimicrobial peptides (Lázár et al., 2018) and that, importantly, some antimicrobial peptides present limited resistance or cross-resistance (Kintses et al., 2019; Spohn et al., 2019).

From a conservative point of view, based on the use of the drugs we already have, it would be desirable to fight AR using evolution-based strategies for developing new drugs or treatment strategies. Regarding this, the exploitation of the evolutionary trade-offs associated with the acquisition of AR, as collateral sensitivity, could allow the rational design of treatments based on the alternation or the combination of pairs of drugs (Imamovic and Sommer, 2013; Gonzales et al., 2015; Barbosa et al., 2018; Imamovic et al., 2018).

In addition to interventions that reduce the selective pressure of antibiotics or that implement new therapeutic approaches, reducing transmission is also relevant to fight infections. The development of drugs or conditions (as certain wastewater treatments) able to reduce mutagenesis or to inhibit plasmid conjugation may also help in reducing the spread of resistance (Thi et al., 2011; Alam et al., 2016; Lin et al., 2016; Lopatkin et al., 2017; Valencia et al., 2017; Kudo et al., 2019). Besides specific drugs to reduce the dissemination of the genetic elements involved in AR, socioeconomic interventions to break the bridges that allow transmission (Baquero et al., 2019) between individuals and, most importantly (and less addressed), between resistance entities (Hernando-Amado et al., 2019) are needed (Figure 3). More efficient animal management, not only allowing less antibiotics use but also reducing animal crowding (and hence AR transmission), as well as improved sanitation procedures, including the universalization of water treatment, will certainly help in this task (Berendonk et al., 2015; Manaia, 2017; Hernando-Amado et al., 2019). Notably, wastewater treatment plants are usually communal facilities where the residues of the total population of a city are treated. Hospitals are the hotspots of AR in a city; hence, on-site hospital (and eventually on-farm) wastewater treatment may help to reduce the pollution of communal wastewater by antibiotics and ARBs (Cahill et al., 2019; Paulus et al., 2019), hence reducing AR transmission.

Concerning trade of goods, it is relevant to remark that, although there are strict regulations to control the entrance of animals or plants from sites with zoonotic of plant epidemic diseases (Brown and Bevins, 2018), there are no regulations on the exchange of goods from geographic regions with a high AR prevalence, a feature that might be taken into consideration for reducing the worldwide spread of AR.

Once ARBs are selected and disseminated, interventions based on the ecological and evolutionary (eco–evo) aspects of AR (Bengtsson-Palme et al., 2018; Lehtinen et al., 2019) should be applied to restore (and select for) susceptibility of bacterial populations, as well as to preserve drug-susceptible microbiomes in humans and in animals (Baquero et al., 2011, 2015). Eco–evo strategies include the development of drugs specifically targeting ARBs. For that, drugs activated by mechanisms of resistance, vaccines targeting high-risk disseminating resistance clones or the resistance mechanisms themselves (Kim et al., 2016; Ni et al., 2017), or drugs targeting metabolic paths that can be specifically modified in ARBs (Baquero and Martinez, 2017) might be useful. The use of bacteriovores such as *Bdellovibrio* to eliminate pathogens without the need for antibiotics has been proposed; although its utility for treating infections is debatable, it might be useful in natural ecosystems (Shatzkes et al., 2016). More recent work suggests that some earthworms may favor the degradation of antibiotics and the elimination of ARBs (Wikler, 2002), a feature that might be in agreement with the finding that ARBs are less virulent (and hence might be specifically eliminated when the worm is present) in a *Caenorhabditis elegans* virulence model (Sanchez et al., 2002; Ruiz-Diez et al., 2003; Paulander et al., 2007; Olivares et al., 2012). However, the information on the potential use of worms for reducing AR in the field is still preliminary and requires further confirmation. Noteworthy, AR is less prone to be acquired by complex microbiomes (Mahnert et al., 2019; Wood, 2019), a feature that supports the possibility of interventions on the microbiota to reduce AR. Among them, fecal transplantation (Chapman et al., 2016; Pamer, 2016) or the use of probiotics able to outcompete ARBs (Keith and Pamer, 2019) has been proposed as strategies for recovering susceptible microbiomes.

## Coda: Antibiotic Resistance, a Pandemic to Which a Global World Seems to be Accustomed

The recent crisis of COVID-19 (Garrett, 2020) resembles the pandemic expansion of ARGs and clearly shows that pandemic outbreaks cannot be solved by just applying local solutions. Further, unless all population is controlled, and comprehensive public-health protocols are applied to the bulk of the population, such global pandemics will be hardly controlled. The case of COVID-19 is rather peculiar, because we are dealing with a novel virus. Very strict interventions have been applied, mainly trying to control something that is a novel, unknown, disease; we have been learning along the pandemic and still ignore what will come further. AR is already a very well-known pandemic affecting humans, animals, and natural ecosystems (Anderson, 1999; Verhoef, 2003). In this case, we have tools that might predict the outcome, and likely because the degree of uncertainty is lower than in the case of COVID-19, we have not applied clear, common, and comprehensive procedures to reduce the spread of AR. It is true that we know the evolution of antibiotics consumption and AR prevalence in several countries, and also interventions, mostly based on social norms, have been applied. Social norms have reduced the unnecessary prescription of antibiotics, or pharmacy sales without prescription, and the use of antibiotics for fattening animals has been banned in several countries, being still allowed in several others. Nevertheless, these actions are not general, and more aggressive, global actions are still needed. Coming back to the COVID-19 example, while the aim of health services worldwide is to detect any possible source of SARS-CoV-2, surveillance of infections (eventually by ARBs) is not universal. In other words, it does not apply to all citizens in all countries. The reasons can be just political such as the inclusion of immigrants in public health services (Scotto et al., 2017) or the consequence of limited financial resources and technical capacity that countries such as those belonging to the LMIC category can face (Gandra et al., 2020). The problem is not only on citizens, because different non-human reservoirs, such as wastewater, drinking water, or freshwater, may jointly contribute to AR dissemination (Hendriksen et al., 2019). In this regard, it is important to highlight that low quality of water is regularly associated to poverty. Universalization of health services, sanitization, access to clean water, and in general reduction of poverty are relevant step-forward elements for reduction of the burden of infectious diseases in general and of AR in particular. The time has come to tackle AR, and this cannot be done just by taking actions at the individual or even country level, but by taking convergent actions across the globe. As stated by John Donne (1923) in his poem, “No Man Is an Island,” written after his recovery from an infectious disease (likely typhus): “No man is an Iland, intire of itselfe; every man is a peece of the Continent, a part of the maine; if a Clod bee washed away by the Sea, Europe is the lesse, as well as if a Promontorie were, as well as if a Manor of thy friends or of thine owne were; any mans death diminishes me, because I am involved in Mankinde; And therefore never send to know for whom the bell tolls; It tolls for thee.”

This reflection on how infectious diseases in general should be faced by the society was published at 1624, but the idea behind still applies nowadays, especially for AR.

## Author Contributions

All authors have contributed to the concept of the review and in its writing.

## Conflict of Interest

The authors declare that the research was conducted in the absence of any commercial or financial relationships that could be construed as a potential conflict of interest.

## Footnotes

1 https://www.who.int/news-room/feature-stories/ten-threats-to-global-health-in-2019
